# Pyjamas, Polysomnography and Professional Athletes: The Role of Sleep Tracking Technology in Sport

**DOI:** 10.3390/sports11010014

**Published:** 2023-01-05

**Authors:** Matthew W. Driller, Ian C. Dunican, Shauni E. T. Omond, Omar Boukhris, Shauna Stevenson, Kari Lambing, Amy M. Bender

**Affiliations:** 1 SIESTA Research Group, School of Allied Health, Human Services and Sport, La Trobe University, Melbourne, VIC 3086, Australia; 2 Sport, Performance, and Nutrition Research Group, School of Allied Health, Human Services and Sport, La Trobe University, Melbourne, VIC 3086, Australia; 3 Centre for Sleep Science, School of Human Sciences, The University of Western Australia, Perth, WA 6009, Australia; 4 Cerebra, Winnipeg, MB R3T 3N9, Canada; 5 Faculty of Kinesiology, University of Calgary, Calgary, AB T2N 1N4, Canada

**Keywords:** actigraphy, wearables, nearables, recovery, orthosomnia, sleep deprivation

## Abstract

Technological advances in sleep monitoring have seen an explosion of devices used to gather important sleep metrics. These devices range from instrumented ‘smart pyjamas’ through to at-home polysomnography devices. Alongside these developments in sleep technologies, there have been concomitant increases in sleep monitoring in athletic populations, both in the research and in practical settings. The increase in sleep monitoring in sport is likely due to the increased knowledge of the importance of sleep in the recovery process and performance of an athlete, as well as the well-reported challenges that athletes can face with their sleep. This narrative review will discuss: (1) the importance of sleep to athletes; (2) the various wearable tools and technologies being used to monitor sleep in the sport setting; (3) the role that sleep tracking devices may play in gathering information about sleep; (4) the reliability and validity of sleep tracking devices; (5) the limitations and cautions associated with sleep trackers; and, (6) the use of sleep trackers to guide behaviour change in athletes. We also provide some practical recommendations for practitioners working with athletes to ensure that the selection of such devices and technology will meet the goals and requirements of the athlete.

## 1. Introduction

Sleep research in athletes has grown considerably over the last decade, allowing us to better understand the nuances, challenges, and issues around how athletes sleep and provide some insights into areas where improvements can be made [1]. Facilitating this, various technologies, tools, and questionnaires to measure, monitor, and screen sleep in athletes have seen an exponential rise in research, practice, and commercial settings [2].

Sleep trackers such as wearables and nearables are one of these tools where we have seen the most significant growth in the sleep-measurement market. These usually include small devices placed on the wrist, finger, head, or chest that record movement as a function of time, with most utilising three-axis accelerometers often in combination with a range of sensors. Sensors can include oximetry, temperature, heart rate/heart rate variability, sound, light, and galvanic skin response, to determine sleep/wake based on a specific algorithm. Unfortunately, the algorithms used by these devices are often proprietary, and very little information is known of the specifics regarding sleep and wake detection. In general, new software or hardware updates and product development to these devices seem to yield improved accuracy, and likely see continual improvements in the accuracy of sleep trackers in the coming years. For now, practitioners, coaches, and researchers working with athletes should be aware of the different sleep tracking devices and their levels of accuracy/validation before deciding on their use. The purpose of monitoring sleep becomes an important consideration (e.g., getting a general idea of sleep patterns in a large squad of athletes vs. testing the efficacy of interventions to enhance sleep). This narrative review will discuss the importance of sleep for athletes, the role that trackers play in gathering information about sleep, the reliability and validity of sleep devices, the limitations associated with sleep trackers, and the use of sleep trackers to guide behaviour changes in athletes.

### 1.1. The Sleep Cycle and Its Importance to the Athlete

**Figure 1:**
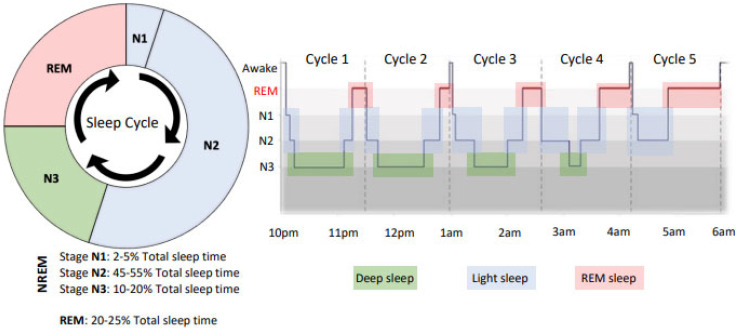
Sleep “architecture”, which shows the typical percentage of time spent in each stage of sleep (NREM 1–3 and REM) and then a sleep hypnogram representing the distribution of these sleep stages across the night, as assessed by polysomnography (PSG). Note that earlier in the night is characterised by a higher proportion of deep sleep (N3) and later in the night (closer to the morning), REM is predominant.

Sleep is an essential psychophysiological process for optimal maintenance of an athlete’s performance, health, and well-being, with the functions of sleep presenting different characteristics depending on the phase of an individual’s sleep. Sleep can be categorised into two main states; non-rapid eye movement sleep (NREM) and rapid eye movement sleep (REM). The NREM sleep is further separated into three stages (N1, N2, and N3), which are considered to be a continuum of sleep depth whereby the human brain enters sleep via NREM Stage N1 and progresses through deeper NREM stages (Stages N2 and N3). Stages N1 and N2 of NREM are considered light sleep and last, on average for 20–30 min [3], whereas Stage N3, “deep sleep” or “slow wave sleep”, lasts for approximately 20 to 40 min in the first sleep cycle [3]. Periods of REM sleep typically follow NREM sleep in the sleep cycle and are characterised by brain activity that resembles wakefulness with low-amplitude, high-frequency electroencephalography (EEG) waves, bursts of rapid eye movements, and muscle atonia preventing the sleeper from acting out their dreams. Indeed, during REM sleep, dreaming is most prevalent, often referred to as “dream sleep”. The three NREM and one REM phases occur cyclically throughout the night, typically taking 70–110 min to complete a full sleep cycle [4] (Figure 1). Humans generally experience 4–7 sleep cycles per night, with different proportions of time spent in each stage throughout the night (Figure 1), depending on factors such as sleep disorders, previous sleep habits, circadian desynchronisation, age, sex, and illness [5]. Typical sleep architecture is characterised by a larger proportion of time spent in NREM in the first half of the night, with REM becoming more frequent in the second half of the night (Figure 1).

It Is well established that the sleep cycle plays a critical role in the psychophysiological recovery of an athlete, where many restorative bodily functions and processes take place. These functions include neuroendocrine, metabolic, and immunological processes that are modulated during various phases of the sleep cycle [6]. An example of this is the essential hormonal reactions that occur before bedtime and throughout the night while we sleep. Growth hormone is vital to physical recovery and regeneration, and essential for muscle development, repair, and bone remodelling [7]. It has been suggested that up to 95% of the daily growth hormone production is released from the pituitary gland in the endocrine system during NREM deep sleep [8]. This is when most of our muscle repair and adaptation takes place following exercise, making it critical for athletes to physiologically adapt to their training [6]. N3 sleep is also thought to be when our immune system is strengthened [9].

The REM sleep cycles are associated with dreaming and are not considered a restful stage of sleep, with EEG traces similar to those found when awake. During REM, the skeletal muscles are atonic and without movement, except for the eyes and diaphragmatic breathing muscles [10]. REM sleep is thought to play a major role in our learning, memory processes, and emotional regulation [11]. We also know that REM sleep is associated with a decrease in norepinephrine levels, a chemical responsible for stress and emotions [12], allowing for a type of “mental recovery”. For the athlete, REM sleep may help them to sharpen their concentration and focus and consolidate procedural memories from a newly learned skill.

### 1.2. Sleep, Athlete Recovery, and Performance

Since 2011, we have seen an exponential increase in research regarding sleep in athletic populations, with over 80% of the overall journal articles published in this field since [1]. This trend speaks to the importance of sleep on the health, recovery, and performance of athletes. Technological advancements are contributing to this growth in research, making it easier to measure and monitor sleep in athletic populations.

Most sleep research in the athletic population has evaluated the effect of sleep restriction or sleep deprivation and the accumulation of sleep debt on various physiological, psychological, and performance outcomes [13]. From this work, it has become clear that sleep loss impairs cognition, learning and memory consolidation, motivation, mood state, immunity, metabolism, and, importantly, athletic performance [2]. However, in comparison, very few previous studies have examined the role of sleep extension and the subsequent benefits for athletes. For example, Mah and colleagues (2011) showed improvements in sprint time and shooting accuracy in 11 collegiate basketball athletes when sleep was extended from 6 h 36 min during a baseline period to 8 h 30 min over a 5–7 week period. Similarly, a study of 29 elite rugby union athletes showed that longer sleep duration during the pre-season may enhance aerobic capacity and body composition over 3 weeks [14].

**Figure 2:**
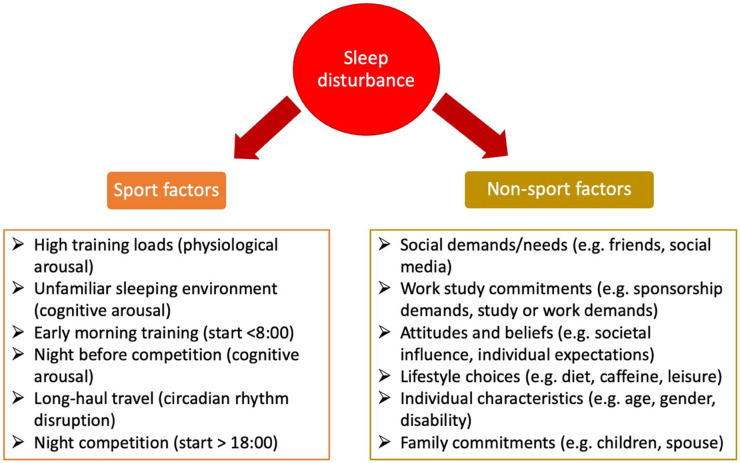
Contributing factors to sleep disturbance in athletes, including both sport and non-sport factors. Adapted from Walsh et al. [2].

Despite the known impact of poor sleep on athletic recovery and performance, elite athletes in the professional era are facing more intensive physical training loads, competition loads, and high levels of mental stress regularly, all of which are factors that could influence sleep disturbances [15]. Alongside this, various studies have identified that athletes may struggle to obtain the recommended 7–9 h of sleep due to different sport- and non-sport-related factors (Figure 2), compromising their recovery [2].

This combination of factors—the realisation of the importance of sleep to athletes, as well as the discovery that many athletes are not obtaining enough sleep—has caused scientists, researchers, and practitioners to turn to non-invasive, cost-effective methods to monitor sleep. This has led to the use of sleep monitoring in the sport and athlete setting, with the hope of gaining valuable information to help improve the sleep patterns of athletes to gain an advantage over the opposition. A survey conducted in 2019 showed that >50% of coaches or practitioners working with elite athletes in Australia monitored their athletes’ sleep, with 75% opting for monitoring using sleep diaries and over 20% using wearable devices [16]. With new and emerging wearable technology, and its improving accuracy for measuring sleep, we will likely see this number increase in the coming years.

### 1.3. Hierarchy of Sleep Assessment

It is essential to know that different methodologies are utilised when measuring sleep, each with its levels of validity and reliability. The application and goal of sleep monitoring will inform which sleep tracking method should be used. Measuring sleep in humans is complex and can take time; therefore, the selection of tools is very important. You must first understand if you are measuring sleep to identify a clinical sleep disorder/problem or if you are trying to quantify sleep behaviours over time. To guide coaches, performance staff, and athletes, our proposed hierarchy of sleep measures may guide decisions on which method to evaluate sleep, including the advantages and disadvantages of each type of technology (Table 1).

### 1.4. Polysomnography and Other Clinical Sleep Tests

Polysomnography (PSG) is considered the gold standard evaluation of sleep measurement that uses a combination of sensors to measure the physiological activity of sleep, including the electrical activity of the brain (electroencephalography (EEG)), eye movements (electrooculography (EOG)), muscle activity of the chin and legs (electromyography (EMG)), and heart rhythm (electrocardiography (ECG)), blood oxygen levels (SpO2), and airflow and breathing (cannula and thermistor) to differentiate the different stages of sleep and breathing pauses that occur during sleep. Although PSG is regarded as the gold standard of sleep measurement, it can be intrusive and expensive.

There are different levels of sleep testing, referred to as Levels I–IV. Level I is the gold standard in laboratory assessment, which utilises PSG and is attended in a sleep laboratory with monitoring from a sleep technician. Levels II also utilises PSG but is unattended, can be self-applied, and is measured in the home. Level III and IV tests are referred to as home sleep apnoea tests and are geared towards measuring sleep apnoea with respiratory-related parameters only and does not include an actual measurement of sleep using EEG.

**Figure 3:**
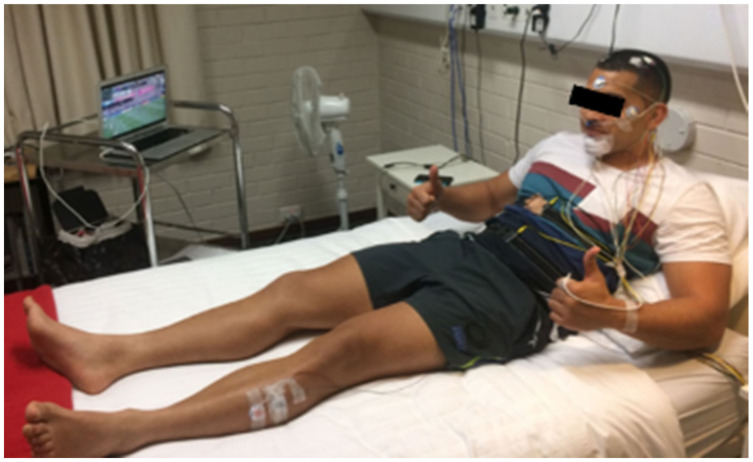
A professional athlete undergoing Level I PSG in a sleep laboratory.

PSG is primarily used for the assessment and diagnosis of sleep disorders, or for a more in-depth assessment of sleep staging and other sleep metrics. As such, individuals with sleep disorders and difficulties are typically prioritised in clinical sleep-lab settings; it can be difficult to use PSG to monitor seemingly healthy populations unless Level II is used, but even so, long-term monitoring is difficult because of the expense of consumables and the intrusiveness of the device (Figure 3). This is perhaps the main reason that we have seen the exponential rise in the sleep wearable business. Many emerging wearable technologies have been developed to address this limitation and propose new ways to measure sleep without the need for full PSG monitoring. In the context of athlete health, PSG does not add a lot of value for measuring sleep longitudinally during a competitive season, during travel, recovery from competition, or in the off-season in athletic populations.

Although PSG is the gold standard, a limited number of studies have used PSG in athletes, and many sports scientists or performance staff do not utilise this methodology to address significant underlying sleep disorders. Despite athletes being highly trained for their chosen sport or skill, the prevalence of sleep disorders and problems is slightly higher compared to the average population, with estimates of sleep disorder prevalence ranging from 20 to 40% [17,18,19].

### 1.5. Wearable Devices

Wearable devices that utilise actigraphy with accelerometers have gained popularity in recent years for measuring sleep and physical activity in various settings from the military, shift workers, athletes, and the general public, with an estimated $81bn end-user spending on wearables globally [20]. In addition, wearable devices represent the majority of the research undertaken in athletic groups to quantify sleep quantity and quality mainly due to the ease of use and an ability to monitor non-invasively for long periods of time.

Typically, these wearable devices are in the form of a watch or ring and are worn on the wrist or finger of an individual. These devices use accelerometers to determine ‘movement’ and/or ‘non-movement’ periods. Accelerometer-based measurements of sleep can be considered in terms of variables that are either ‘directly’ measured by the device or required to be manually entered (e.g., time at lights out, time at sleep onset, wake after sleep onset, and time at wake) or variables that are derived from these measures (e.g., sleep latency, sleep duration and sleep efficiency). In addition, newer actigraphy devices have more commonly utilised the use of photoplethysmography (PPG), which emits light onto the skin and evaluates the change in light absorption to obtain heart rate and heart rate variability. The introduction of PPG into wearables has increased the accuracy of the readings compared to devices without [21].

### 1.6. How Valid and Reliable Are the Measures Gained from Sleep Wearables?

The rapid growth of the field of wearables reflects a motivation to find an appropriate device for long-term monitoring of sleep. The market is full of devices claiming to easily track sleep on a nightly basis, and many have shown initial promise for some measurements of sleep. First, however, it is important to recognise what these devices are currently capable of achieving and where they still require advancements. The development of wearable devices is complicated by a balance between accuracy for use in clinical, consumer, or research settings and with cost and practicality for widespread and long-term use [22].

Comparisons between wearable devices and more standard measures, such as PSG, highlights room for improvement. These devices have high sensitivity in detecting sleep periods (91–96%) but lower specificity in detecting wake, ranging from 18% to 80% across studies averaging around 50% [23,24,25,26,27,28,29,30]. The challenge with specificity is partially due to these devices being indirect measures of sleep that do not record brain activity. For example, in detecting sleep onset, devices that base their prediction of sleep on the wearer being immobile underestimate sleep onset latency compared to polysomnography [22]. This result is expected with movement to detect sleep, as the user will cease movement before the onset of sleep. In addition, independent company algorithms are often proprietary to the company, making them challenging to objectively assess. However, recent commercial devices are now performing more similar to research-grade actigraphy for the detection of sleep and wake, which, as mentioned, has challenges in detecting wakefulness [23,24].

There have been studies to compare the performance of different wearable sleep trackers against each other. As has been found previously, the devices were generally sensitive in their detection of sleep but low in their specificity, resulting in an overestimation of sleep as they miss the detection of wake [23,24,28,31]. Regarding reliability, when wearing two of the same devices simultaneously, the agreement for sleep is generally high [32,33]. Comparison between different placements of devices has also been performed, with one study comparing a chest-worn sensor that incorporates body position and acceleration with PSG and wrist [34]. Using these additional signals on the body as opposed to the wrist accelerometry had a better comparison to PSG for the detection of sleep and wakefulness. Previous research has also suggested that the wrist that an actigraph is worn on (dominant or non-dominant) may not matter for sleep outcome measures [35].

With the addition of other signals, such as heart rate measured with photoplethysmography, wearable devices are now aiming to identify different sleep stages, including light sleep, deep sleep, and REM. However, in a consensus statement from an expert panel of sleep researchers in 2020, it was claimed that there was insufficient evidence to support sleep staging in wearable devices [27], though this is likely to change in the future as wearable devices evolve.

### 1.7. Wrist-Worn Wearables

Wrist-worn devices are perhaps the most common sleep wearable used in research and athlete settings. Indeed, most smartwatch brands now include in-built sleep monitoring features. These devices are readily accessible, and if the goal is to measure changes in overall sleep behaviour rather than stage-specific data, then these well-supported easy-to-use devices may be preferential. Devices termed “research-grade actigraphy” have been the most commonly used devices in the athlete research setting up until more recently. These devices include the Actiwatch, Actigraph, the Mini-Mitter actigraph, and the MotionWatch actigraph. With the rise in commercial wrist-worn devices, we have seen the likes of Whoop, Readiband, Fitbit, Apple Watch, Garmin, Samsung, Polar, and many other devices enter the market and become more commonly used in both sport and research settings. With the increased cost of “research grade actigraphy” and the loss of support (the Actiwatch will be discontinued in 2022, and support discontinued in 2024), it is probable that researchers may take up more commercial and proprietary methods of sleep monitoring. The first generation of wrist-worn wearables used accelerometer sensors in isolation to determine sleep and wake, which makes them somewhat limited as they measure only movement and non-movement periods [36]. However, the new generation of wrist-worn wearables tracks sleep based on movement, skin temperature, pulse oximetry, heart rate, and heart rate variability [36]. These advances in sensor technologies allow the prediction of sleep stages and may even raise red flags for potential sleep disorders.

### 1.8. Finger-Worn Wearables

Another alternative for wearable devices is those that are worn on the finger, often referred to as smart rings. With the rise in commercial finger-worn devices, we have seen the likes of Oura, THIM, Motiv, GO2SLEEP, and others join the market and start gaining popularity in the sport and research setting. The Oura ring uses a combination of sensors, including motion, body temperature, heart rate, heart rate variability, and pulse wave variability amplitude, to measure sleep and derive sleep staging information [37]. According to de Zambotti [38], the Oura ring measured sleep with 96% sensitivity and agreement of 65%, 51%, and 61% in measuring light, deep, and REM sleep, respectively. Recently, Altini and Kinnunen [39] demonstrated that the Oura ring measured sleep-wake with a 94% accuracy based on accelerometer sensors and a 96% accuracy when based on a combination of accelerometer, HRV, temperature, and circadian features with machine learning techniques. However, for sleep-stage detection in the same study, 57% accuracy was reported for the simple accelerometer-based model, and 79% accuracy was reported for the full model that contained ANS-derived and circadian features. For the THIM ring (which uses a tri-axial accelerometer sensor), Scott et al. [40] reported a lower sensitivity (0.91) and a higher specificity (0.59) compared to Actiwatch (0.95 and 0.35, respectively) and Fitbit (0.98 and 0.32, respectively) devices.

Additionally, no significant differences were reported between the THIM ring and PSG in measuring total sleep time, sleep onset latency, wake after sleep onset, or sleep efficiency. The GO2SLEEP ring uses heart rate, blood oxygen level and PPG sensors to monitor sleep [38], but to the authors knowledge, no validation studies exist for the GO2SLEEP ring or the Motiv ring against PSG. While finger-worn wearables show a lot of promise and convenience, further validation studies are required, especially on their accuracy in detecting sleep stages.

### 1.9. Clothing-Based Sleep Monitoring

A relatively novel development in the sleep wearable world comes in the form of “smart pyjamas”. In particular, “Phyjamas” (named due to their proposed physiological measurement) are a fabric-integrated tracker containing a network of fabric-based sensors that monitor and provide the key metrics of sleep [41]. Using sensor-augmented loose-fitting sleepwear, ballistic movements, respiratory rate, and heart rate can be measured. The electromechanical sensors include four piezoresistive pressure sensors to detect constant pressures and one triboelectric sensor to detect quick changes in pressure. While these smart pyjamas have been validated for sleeping positions [42], they are yet to be validated for measuring important sleep metrics. This may be an area of future technological enhancements in sleep monitoring.

### 1.10. Head-Worn Sensors

Recent technology has focused on head-worn sensors for enhanced sleep monitoring that considers EEG/EOG/EMG signals alongside other measures used by other wearables. For example, the Somfit (Compumedics, Australia) is a device attached to the forehead using a single gel adhesive strip that measures EEG and EOG/EMG (derived from EEG signal), oxygen saturation, heart rate, and heart rate variability to determine sleep and sleep staging. Compared to PSG for the two-state categorisation of sleep/wake, Somfit correctly identified 92% of sleep epochs and 57% of wake epochs with a kappa value of 0.48, which indicates a moderate level of agreement [43]. Compared with PSG for multi-state categorisation of sleep/wake, Somfit correctly identified 65% for deep sleep (N3), and 58% for REM sleep, with a kappa value of 0.52, which indicates a moderate level of agreement for sleep staging [43].

Similarly, the Dreem headband is another common head-mounted sleep device that uses five EEG electrodes (O1, O2. FpZ, F7, F8) to measure brain cortical activity and a 3D accelerometer to monitor position, movement, and breathing frequency, and a pulse oximeter to monitor heart rate [44]. In a validation study performed by the manufacturers, the Dreem headband resulted in an 83% overall scoring accuracy with PSG across five stages (the four sleep stages and wake) and 74% accuracy with PSG for wake epochs alone [44].

Results from the mentioned studies indicate that these devices might be an option for field-based assessment of sleep and may be used as an alternative to PSG in estimating two-stage sleep [43]. However, further independent validation of these head-worn devices is needed to determine their accuracy for sleep staging.

### 1.11. Nearables

While not technically a “wearable” technology, another emerging area of sleep tracking devices is the use of nearables. Unlike wearables, nearables detect sleep from a distance and are generally situated under/on the mattress or a bedside stand. Such devices usually include ballistocardiography vibration (for respiratory and heart rate, stroke volume), light, sound, humidity, temperature, and motion sensors to estimate sleep [45]. Products that are located under the mattress (e.g., EarlySense, EmFit, and Withings Aura) or on the mattress (e.g., Beddit, Eight mattress cover, and RestOn) use sensors such as ballistocardiogram, respiration, and motion sensors [46], while devices such as the SleepScore Max and ResMed S+ are products located near the bed (i.e., on your bedside table) and use a combination of light and sound sensors to estimate sleep [46]. When compared to PSG for the two-state categorisation of sleep/wake, EarlySense, SleepScore Max, and ResMed S+ showed a very high sensitivity (all ≥0.93) with specificities of 0.47, 0.50, and 0.51, respectively [23]. In addition, the Beddit device identified total sleep time, sleep efficiency, sleep onset latency, and wake after sleep onset with an excellent agreement compared to PSG (all ICC values  ≥  0.92) [47]. However, when compared with PSG, the EmFit failed to recognise sleep stages, and for the two-state categorisation of sleep/wake, the EmFit resulted in a low agreement, with a kappa value of 0.13 [48]. Although nearable devices could be a good choice for individuals who dislike wearables while sleeping, many of them still lack validation studies against PSG.

### 1.12. Sleep Questionnaires and Diaries

Another common sleep monitoring method is sleep-related questionnaires and sleep diaries, commonly used to assess sleep in athletes. Although there are many questionnaires developed for the general population or those with sleep disorders (e.g., the Epworth Sleepiness Scale or Stanford Sleepiness Scale for daytime sleepiness, Insomnia Severity Index for insomnia, Berlin Questionnaire and STOP BANG for sleep apnoea, Morningness and Eveningness Questionnaire for diurnal preference and the Pittsburgh Sleep Quality Index for sleep quality), they may not be specific or relevant to athletes.

This is where newly developed tools such as the Athlete Sleep Screening Questionnaire (ASSQ) [49], which screens athletes for clinically relevant sleep disturbances, or the Athlete Sleep Behaviour Questionnaire (ASBQ) [50], which targets athlete-related sleep behaviours may be beneficial in use with athletic populations. Sleep diaries may also provide useful insights into the sleeping patterns of athletes or may be a useful tool to sit alongside a sleep wearable. In general, athletes tend to overestimate their sleep duration by 30 min to 1 h compared to validated wearable devices [51].

### 1.13. Sleep Metrics Provided by Sleep Trackers

Sleep-related trackers can provide a plethora of sleep and wake-related variables. The challenge is working out which are worth using and which are not. Many commercially available sleep trackers have their proprietary algorithms with novel scores such as sleep and readiness ratings. However, caution should be exercised when using such data to infer readiness, alertness, or recovery with elite athletes, as these measures have not necessarily been validated and are subject to high levels of inter-individual variability. Therefore, it would be beneficial to clarify the validity of such metrics before using them with athletes, as it may lead to negative reinforcement. The recommended sleep measures to be utilised when analysing sleep/wake behaviours are described in detail below in Table 2.

### 1.14. How Can We Use Data to Guide Changes in Sleep?

To use the data collected by a sleep tracker, expertise is needed to interact and interpret the right data to make sense of how it can be used to improve sleep. As mentioned above, sleep wearables may be accurate for sleep duration but struggle with accuracy when analysing sleep stages. Due to the ease of use of these systems, it is common for athletes to become hyper-focused on their sleep output from their devices and become stressed about the staging. As a researcher or practitioner, our role in using these devices must be one of relaying the caveats about their capabilities and limitations. Sleep onset latency (i.e., the time it takes to fall asleep) is another example where most wearables use movement, or lack of it, to determine sleep. As such, we know that it is likely underestimated because we lay still for a few minutes prior to falling asleep. However, if it is a situation where their overall sleep duration is lacking across a few days to weeks at a time, we can more easily use the data as an awareness tool to elicit behaviour change to try to encourage them to get more sleep because the accuracy of sleep duration is solid. Generally, it is recommended to observe rolling averages of sleep duration in periods such as 14, 21, or 28 nights. Understanding these limitations can help improve how you inform the athlete about their data. Using these devices as a comparative guide, rather than accurate sleep staging, may be more beneficial to the athlete, as seeing overall sleep length improving may be the goal, rather than trying to increase time spent in various stages in this sense, baseline readings are essential.

We also need to be aware of the feedback being given from the device and how that can impact an athlete’s cognition and performance. One study in non-athletes with insomnia found that negative sham feedback, where they were wrongly told that they slept poorly, led to impaired cognition and more sleepiness, compared to the positive feedback group, which showed better mood and alert cognition from rising time and overall decreased fatigue [52]. Similarly, Rahman et al. [53] conducted a study on healthy participants whereby participants were deceived on whether they received 8 h or 5 h of sleep per night via a bedside clock rigged to run fast or slow. Those who believed they slept for 8 h, even when it was only 5 h, performed significantly better on the reaction time task than those who perceived to have only 5 h of sleep [53]. If the information coming from the device is not accurate, to begin with, in the negative direction, this could be damaging to daytime functioning, especially if the feedback is on an important competition or training day. Certain wearable platforms allow feedback to be turned off, which is advantageous as it relates to important games or competitions.

Researchers and practitioners have also sought to use sleep data to measure the impact of sleep hygiene/behavioural education on changes to sleep. Sleep hygiene education sessions tend to focus on understanding sleep and what happens when you sleep, the common sleep problems faced by athletes, the importance of sleep for athletic recovery, and strategies to enhance sleep. In this context, several studies have evaluated the impact of sleep hygiene education on sleep quality and quantity [54,55,56,57]. For example, Fullagar et al. [54] reported that an acute sleep hygiene strategy (e.g., cool temperature room ~17 °C, eye masks and ear plugs, no light or technological stimulation permitted ~15–30 min prior to bedtime) following a late-night soccer match in highly trained amateur soccer players resulted in greater sleep duration in players who completed the sleep hygiene strategy (6:09 ± 0:43 h: min) compared to those without any assistance or recommendations for sleep (4:30 ± 0:27 h: min). Driller et al. [57] reported that personalised sleep hygiene education (a 50 min group session and a 30 min one-on-one session) led to improvements in two sleep questionnaires (ESS and PSQI) and sleep latency (−29 min), sleep efficiency (+5%), and sleep onset variance (−28 min) in male cricket athletes over a 3-week period. In a longer study, Caia et al. [56] investigated the impact of two 30 min sleep hygiene education seminars delivered over successive weeks on the sleep of professional rugby league athletes during 10 weeks of the competitive season. The study found that the initial sleep hygiene education seminar led to an increase in sleep duration by ~20 min compared to baseline. However, one month later, sleep behaviour and metrics returned to baseline values, suggesting the importance of continuing sleep education in athletes [56].

### 1.15. Limitations of Using Wearables in Athletes

The rapid uptake of wearables to measure sleep in the athletic setting is not without limitations. While using such technology may have favourable outcomes for some individuals, providing an opportunity to identify various sleep metrics and patterns that may be improved via behavioural changes, for others, it may become an unhealthy obsession. In the general population, up to 50% of adults in the United States of America have considered purchasing a sleep tracking device. For the use of their own sleep tracking devices, increasing numbers of patients have been seeking treatment in clinical sleep laboratories based on their sleep data and concerns about their reported sleep quality or quantity from their devices. It may be that some personality types, for example, perfectionists or type A personalities, will strive in the quest for “perfect” sleep numbers, which of course, is a futile endeavour and may further exacerbate any sleep issues. This phenomenon has been termed “orthosomnia”, with “ortho”, meaning straight or correct, and “somnia”, meaning sleep, whereby individuals are preoccupied or concerned with improving or perfecting their wearable sleep data [58]. The trust that some individuals put in the accuracy of their data from sleep wearables is somewhat concerning, and as discussed earlier in this Section, not all wearables result in acceptable levels of agreement with gold standard measures of sleep, such as PSG, especially for the time spent in different phases of the sleep cycle. Furthermore, some individuals become focused on improving the time spent in certain phases of sleep (e.g., N3 or REM) based on their sleep wearable data, with the aim to improve sleep quality while discarding the importance of total sleep duration.

As human beings, we are reasonably adept at judging the quantity of our sleep without seeing any data to guide us. Research in athletes has shown that they could predict their total sleep duration within around 20 min compared to data obtained with wrist actigraphy [51]. Therefore, this leads us to question the need to measure sleep duration using wearable technology, as sleep diaries may suffice. Indeed, depending on the goal of any sleep monitoring with athletes, sleep diaries can still provide enough valuable information concerning sleep patterns (bed and wake times) and durations of sleep. However, what we cannot seem to judge as accurately is the time it takes us to fall asleep, the number of times we wake up during the night, and the overall quality of our sleep. This is where wearable sleep devices may be more useful, but as discussed, not all devices can be trusted for such measures, and practitioners working with athletes should be selective in the devices they use and the goals of monitoring sleep in this population.

Many commercially available sleep trackers targeted towards the sport and athlete market include various “recovery” or “readiness” scores built into their proprietary algorithms, based largely on the measured sleep data. These scores will often tell an athlete how recovered they are based on the data and may even prescribe what training should be performed based on their score. For example, a poor night of sleep may result in a low readiness score, subsequently telling the athlete to take it easy or take a day off today. While this may provide useful information for some users, given that the algorithms used to derive these scores are usually patented, it makes it difficult for scientists and researchers to interrogate such metrics, leading to questions about the usability and validity of such scores, especially in the elite athlete setting. Again, depending on the personality of the athlete and the trust in the device, seeing a low readiness score on the morning of an important competition; for example, an Olympic final may be negative for the motivation and performance of the athlete. Therefore, caution should be taken on using these scores to inform training and performance.

### 1.16. Data Ownership and Privacy

With many of the sleep devices currently used to measure sleep in athlete populations, data are often sent to an external device or a central cloud-based module for further processing. Sometimes, this will involve software downloaded to a computer, or it will be processed using third-party online software. While there has been an exponential increase in the published literature describing the validity and accuracy of sleep monitoring devices, to the author’s knowledge, very little has been described in terms of the security and privacy of the collected data. Given that sleep data can be considered a type of medical record, care needs to be taken about data ownership and privacy. Further, sleep data and information of high-profile athletes may be of interest to the general public, so ensuring that this data will not be compromised or leaked is of utmost importance. One way to ensure anonymity when measuring sleep in athletes is to assign each athlete a code and not use their name or any other information that might make them easily identifiable.

### 1.17. Practical Recommendations for the Use of Sleep Wearables in Sport

In general, when assessing sleep, tracking devices should be worn for an absolute minimum of 7 nights to ascertain sleep/wake behaviours, and depending on the capability and battery life of the device, it is recommended that they are used for even longer periods (e.g., 2–3 weeks) to gather useful information on typical sleep patterns. They can also be a preliminary screening method for certain sleep disorders or problems such as circadian rhythm disorders, social jet lag, and insomnia [59]. In addition, they may assist in objectively determining the effects of travel fatigue and jet lag and support effective real-time jet lag minimisation strategies when travelling if they contain an automated scoring algorithm. Therefore, it is important to outline with your athletes what the purpose of using the sleep device is and the overall goal of what you are.

## 2. Conclusions

Technological advancements over the past few years have already resulted in the gap between research-grade sleep monitoring that you typically find in a sleep clinic and commercially available sleep trackers becoming a lot narrower [23]. Sleep tracking has become a big business, and investment in this area will see great technological advances in the coming years. Advances are likely to include improved wearables’ accuracy, reliability, and validity, while also becoming minimally invasive and easy to use for the wearer. New wearable devices placed in or on other areas of the body (e.g., in-ear wearables, or “earables”) are also starting to emerge [60]. While there is insufficient evidence for commercially available wearables to support accurate sleep staging at this stage, the investment and technological advancements that are occurring are likely to change this in the future [27].

Small, wireless, multi-channel, wearable sensors will become commonplace, and the accuracy of such sensors will become so advanced that full PSG monitoring in a sleep laboratory may not be required in the future. However, at this point in time, PSG still plays an important role, especially in the diagnosis of sleep disorders, and until the performance of less-invasive technology supersedes PSG, it remains a critical tool. Furthermore, just because the technology will improve in terms of its accuracy, there are still many other considerations before making sleep monitoring a standard practice when working with athletes. As discussed in this review, the goals of sleep monitoring and consideration of the individual athlete need to be factored in before implementing widespread monitoring across squads of athletes.

## Figures and Tables

**Table 1:** Sleep measurement tools, with advantages, disadvantages and use-case examples.

Assessment Tool	Description	Advantages	Disadvantages	When to Use	Examples
**Polysomnography (PSG) Level I**	Gold standard for the assessment of sleep	Used to diagnose a sleep disorder	Not suitable or designed for longitudinal measures of sleep in athletes	Used to assess for the prevalence of sleep disorders such as sleep apnoea	Compumedics Somte, Philips Respironics Alice 6, Nahon Kohden PSG-1100
		Used for Multiple Sleep Latency Test and Maintenance of Wake Test	Not for evaluation of pre- versus post-interventions in sleep behaviours	Generally used for one to two nights	
		Method for determining sleep architecture (sleep stages)	Expensive and requires specialist expertise	Laboratory-based research	
			Intrusive and can be a bad night’s sleep for the individual	When investigating interventions that might influence sleep architecture	
			Is not indicative of sleep behaviours		
**Polysomnography (PSG) Level II**	Used for home-based assessment of sleep disorders	Method for determining sleep architecture (sleep stages)	Not suitable for everyday use, but can do multiple nights in the home	Used to assess for the prevalence of sleep disorders such as sleep apnoea	Cerebra Sleep System, Sleep Profiler
		Allows the person to sleep in their bed	Expensive and requires specialist expertise	When investigating interventions that might influence sleep architecture	
		Unattended, and some devices are self-applied	Intrusive and can be a bad night’s sleep for the person	For high-profile elite athletes that do not wish to attend a laboratory	
		Objective measure of sleep and sleep disorders			
**Home Sleep Apnoea Testing (HSAT) Levels III–IV**	Used for home-based assessment of sleep apnoea	Allows the person to sleep in their bed	Not suitable for everyday use, but can do multiple nights in the home	For sleep disorder assessments in different environments, e.g., at altitude	Remmers Sleep Recorder, ApneaLink
		Objective measure of sleep-disordered breathing	Not for evaluation of pre- versus post-interventions in sleep behaviours	For high-profile elite athletes that do not wish to attend a laboratory	
		Do not have to attend a sleep laboratory	Expensive and requires specialist expertise	When pre-test probability deems Levels III–IV	
			Intrusive and can be a bad night’s sleep for the person		
			Does not measure sleep using EEG		
**Wearables**	Wrist- or finger-worn actigraphy devices utilising accelerometery and/or sensors to determine sleep/wake periods and behaviours	Allows longitudinal measures of sleep (over a season)	Lack of validation for some devices	Can be used anytime; during a playing season, pre-season, or off-season	Readiband, Fitbit, ActiGraph, Whoop, ActiWatch, Oura, Garmin, Apple Watch
		Physical activity trackers (FDA approved)	Not classified as medical devices	Recommended for at least 2 weeks to establish sleep behaviours	
		Used for assessment of circadian rhythm disorders, such as jet lag, shiftwork disorder, and phase delay	Does not assess sleep-related breathing disorders or movement disorders		
		Relatively low cost and easy to use	Difficult to aggregate data, may require additional cost to aggregate and analyse		
		Promote athlete and performance staff interaction and discussion	May not provide accurate representation of sleep stages		
		Supports further evaluation or PSG for sleep disorders	Some devices require a scientist to score the data		
		Many have automated scoring algorithms that can be instantly provided to a smartphone	Some devices require a sleep diary to completed in conjunction with wearing the device		
		Minimally invasive	Algorithms and scores are not openly reported/validated		
**Nearables and Smartphone Applications**	Devices placed in proximity of the person sleeping, in the bed, or near the bed	Easy to use and often preferred to use instead of wearables or PSG	May measure sleep from bed partner		Beddit, Resmed+, Sleep Score, Sleep cycle applications
		Can increase awareness of sleep and sleep problems	May be impacted by pets in the bedroom		
		Device does not have to be worn—minimally invasive	Lack of validation on many smartphone applications		
**Validated Sleep-Related Questionnaires**	Used to determine the potential prevalence of sleep disorders and problems	Low cost, easy to use, and requires no training or expertise	Not a clinical diagnosis and requires further assessment	Can be used anytime; during a playing season, pre-season, or off-season	Athlete Sleep Behaviour Questionnaire, Athlete Sleep Screening Questionnaire, PSQI, ESS, Berlin, ISI, Sleep Hygiene Index.
		Used as a baseline of sleep behaviours and problems	Some not specific to the challenges that athletes face		
		Can be used as a precursor for the potential prevalence of a sleep disorder			
		Used for organisational and demographic reporting and potential health promotion or interventions			
**Sleep Diaries**	Self-reported reflective sleep timing based upon recall	Low cost, easy to use, and requires no training or expertise	People tend to overestimate sleep duration, underestimate sleep latency and wake time	Can be used anytime; during a playing season, pre-season, or off-season	
		Well used in the research literature	Long-term adherence to filling out sleep diaries is generally poor		
		Can provide interesting information on sleep timings (time at sleep and time at wake)	Difficult for coaches and leaders to take actions based upon this data as highly variable		

**Table 2:** Common measures from sleep tracking devices.

Sleep Measure	Acronym	Units	Abbreviated	Measurement	Description
Sleep Onset Latency	SOL	Minutes	mins	Derived	Number of minutes from Time at Lights Out to Time at Sleep Onset
Sleep Onset Time	SOT	Time of day	hh:mm	Directly measured	Time of day when the first epoch of sleep occurs between Time at Lights Out and Time at Wake
Total Sleep Time	TST	Hours and Minutes	h:min	Derived	Number of minutes from Time at Sleep Onset to Time at Wake, minus number of minutes awake (WASO)
Wake After Sleep Onset	WASO	Minutes	mins	Directly measured	Number of minutes awake after Time at Sleep Onset
Wake Time	WT	Time of day	hh:mm	Directly measured	Time of day when awake with no further Sleep Duration
Sleep Efficiency	SE	Percentage	%	Derived	Sleep Duration divided by Time in Bed multiplied by 100
Time in Bed	TIB	Hours and Minutes	h:min	Derived	The total time spent in bed, from Time at Lights Out to Time at Wake
Sleep Onset Variance	SOV	Hours and Minutes	h:min	Derived	Can be calculated using standard deviations or by using the sleep regularity index (SRI)
Wake Variance	WV	Hours and Minutes	h:min	Derived	Can be calculated using standard deviations or by using the sleep regularity index (SRI)

## Data Availability

Not applicable.
